# Interventional Radiology for Delayed Post-Pancreatectomy Hemorrhage: A Case Report and Review of the Literature

**DOI:** 10.7759/cureus.94945

**Published:** 2025-10-19

**Authors:** Ruchika Patra, Sidra Baig, Dakshin Meenashi Sundaram, Riten Bhadreshkumar Patel, Anushka Mehta, Uzoh Chidimma Judith, Pooja Patel, Sajjal Mahmood, Harshini Malisetty, Prachi Dawer

**Affiliations:** 1 Department of Burn and Plastic Surgery, All India Institute of Medical Sciences, Bhubaneswar, Bhubaneswar, IND; 2 Department of Internal Medicine, Dr. Vizarath Rasool Khan (VRK) Women’s Medical College, Hyderabad, IND; 3 Department of Internal Medicine, Employees' State Insurance Corporation (ESIC) Medical College and Post Graduate Institute of Medical Sciences and Research (PGIMSR), Chennai, IND; 4 Department of Internal Medicine, Penza State University, Penza, RUS; 5 Department of Internal Medicine, Gullas College of Medicine, Cebu, PHL; 6 Department of Internal Medicine, National Pirogov Memorial Medical University, Vinnytsia, UKR; 7 Department of Internal Medicine, Smt. B.K. Shah Medical Institute and Research Centre, Vadodara, IND; 8 Department of Internal Medicine, Sheikh Zayed Medical College (SZMC) Rahim Yar Khan, Rahim Yar Khan, PAK; 9 Department of Medicine, Government Medical College, Mahabubnagar, Mahabubnagar, IND; 10 Department of Neurosurgery, University College of Medical Sciences, New Delhi, IND

**Keywords:** angiography, delayed hemorrhage, embolization, interventional radiology, pancreaticoduodenectomy, post-pancreatectomy hemorrhage, pseudoaneurysm

## Abstract

Post-pancreatectomy hemorrhage (PPH) is a life-threatening complication of pancreatic surgery associated with high mortality. The International Study Group of Pancreatic Surgery (ISGPS) classifies PPH as early (<24 hours) or delayed (>24 hours), with delayed hemorrhage often resulting from vascular erosion, pseudoaneurysm formation, or anastomotic ulceration.

We report a case of a 52-year-old male who underwent a classical pancreaticoduodenectomy (Whipple’s procedure) for periampullary adenocarcinoma. The early postoperative course was uneventful until postoperative day (POD) 6, when the patient developed a sentinel bleed, presenting as hematemesis and melena, followed approximately two hours later by hemodynamic instability (BP 80/50 mmHg). Laboratory evaluation revealed a sharp drop in hemoglobin from 11.2 g/dL to 6.4 g/dL, with normal coagulation parameters.

Emergency digital subtraction angiography demonstrated a pseudoaneurysm near the gastroduodenal artery (GDA) stump with active contrast extravasation. Selective transcatheter arterial embolization (TAE) using microcoils and embolic agents achieved immediate angiographic success; however, despite aggressive resuscitation and vasopressor support, the patient’s condition deteriorated, and he succumbed to massive hemorrhage and its complications. There was no evidence of pancreatic leak or intra-abdominal sepsis.

This case highlights the catastrophic potential of delayed PPH, where sentinel bleeding serves as an early warning sign. Prompt recognition, urgent angiography, and a multidisciplinary approach are critical to improving patient outcomes, with interventional radiology offering a minimally invasive, potentially lifesaving alternative to high-risk re-exploratory surgery.

## Introduction

Complications following pancreatic surgery, such as anastomotic leak, postoperative pancreatic fistula formation, hemorrhage, and delayed gastric emptying, remain major causes of postoperative morbidity and mortality. Among these, hemorrhage is particularly perilous, with reported mortality rates reaching up to 40-50% [1]. The International Study Group of Pancreatic Surgery (ISGPS) classifies post-pancreatectomy hemorrhage (PPH) into early (<24 hours, acute) and delayed (>24 hours, chronic) types, based on the timing of onset after surgery [1]. While early hemorrhage is usually attributed to technical factors or inadequate intraoperative hemostasis and often requires surgical re-exploration, delayed hemorrhage generally occurs due to vascular erosion secondary to intra-abdominal sepsis, pancreatic or biliary fistula, anastomotic ulceration, or the formation of an arterial pseudoaneurysm [2,3]. Common arterial sites prone to delayed PPH include the gastroduodenal artery stump (up to 60%), common hepatic artery (approximately 20-25%), splenic artery (10-15%), and superior mesenteric artery branches (less than 5%) [4,5]. With a mortality rate approaching 50%, delayed PPH remains one of the most severe and life-threatening complications following pancreaticoduodenectomy or distal pancreatectomy. In recent years, interventional radiology has emerged as a first-line, minimally invasive, and organ-preserving approach in the multidisciplinary management of delayed hemorrhage, reducing the need for high-risk surgical re-exploration and improving overall survival outcomes.

## Case presentation

A 52-year-old male with a known history of type 2 diabetes mellitus and hypertension, well controlled on oral medications, presented with painless progressive jaundice, pruritus, and weight loss of two months’ duration. Contrast-enhanced computed tomography (CECT) of the abdomen revealed a periampullary mass measuring 2.8 × 2.3 cm without vascular invasion or distant metastasis. Endoscopic biopsy confirmed periampullary adenocarcinoma. The patient subsequently underwent a classical pancreaticoduodenectomy (Whipple’s procedure) with duct-to-mucosa pancreaticojejunostomy, hepaticojejunostomy, and gastrojejunostomy reconstruction.

The surgery was uneventful, and the patient was managed postoperatively in the ICU for 48 hours before transfer to the surgical ward. The patient received standard postoperative care, including broad-spectrum antibiotics, thromboprophylaxis, glycemic control, and early enteral nutrition. The surgical drain initially yielded 80-100 mL of serous fluid daily. Drain fluid amylase on postoperative day (POD) 3 was 45 IU/L, not suggestive of a pancreatic fistula. No intra-abdominal collections were detected on ultrasound.

On POD 6, the patient experienced two episodes of hematemesis and passage of melena, followed by hypotension (BP 80/50 mmHg) and tachycardia (heart rate (HR) 120 bpm) within two hours of the initial bleed. Given the hemodynamic deterioration, this was considered a major GI hemorrhage, not a classical sentinel bleed, which is typically minor and self-limiting. The drain output on the same day became altered and blood-stained (approximately 250 mL) compared to previous serous output. Laboratory results showed a decline in hemoglobin from 12.1 g/dL preoperatively to 10.8 g/dL on POD 1, and to 6.4 g/dL on POD 6, while the coagulation profile and platelet count were within normal limits.

**Figure 1:**
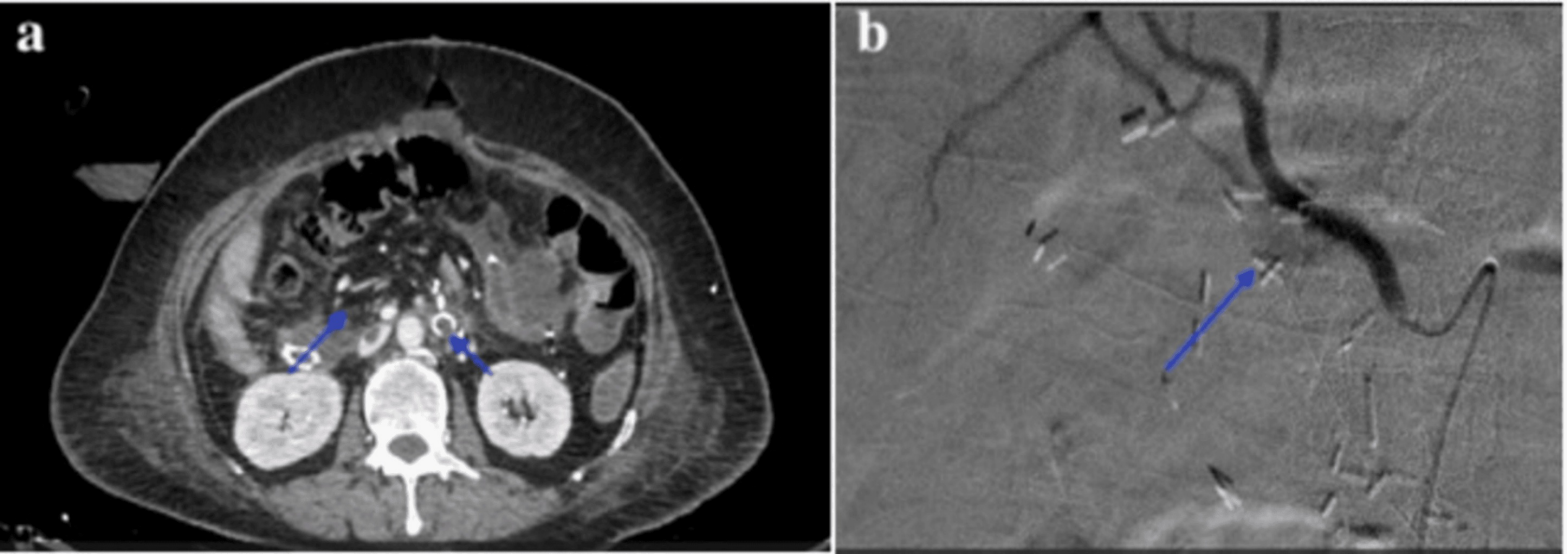
(a) Axial contrast-enhanced CT image showing a pseudoaneurysm (yellow arrow) near the gastroduodenal artery stump with contrast extravasation. (b) Digital subtraction angiography (DSA) image confirming active contrast extravasation from the GDA stump pseudoaneurysm.

The patient was shifted to the ICU and received two units of packed red blood cells and one unit of fresh frozen plasma. Emergency CECT angiography demonstrated a pseudoaneurysm arising from the stump of the gastroduodenal artery (GDA) with active contrast extravasation into the peripancreatic region (Figure 1a). Right common femoral artery access was obtained, and selective celiac and common hepatic artery angiography confirmed the pseudoaneurysm at the GDA stump (Figure 1b). Super-selective catheterization was performed using a 2.7 Fr microcatheter, and the pseudoaneurysm was embolized with platinum microcoils (2 mm × 6 cm) and polyvinyl alcohol (PVA) particles (300-500 μm).

**Figure 2:**
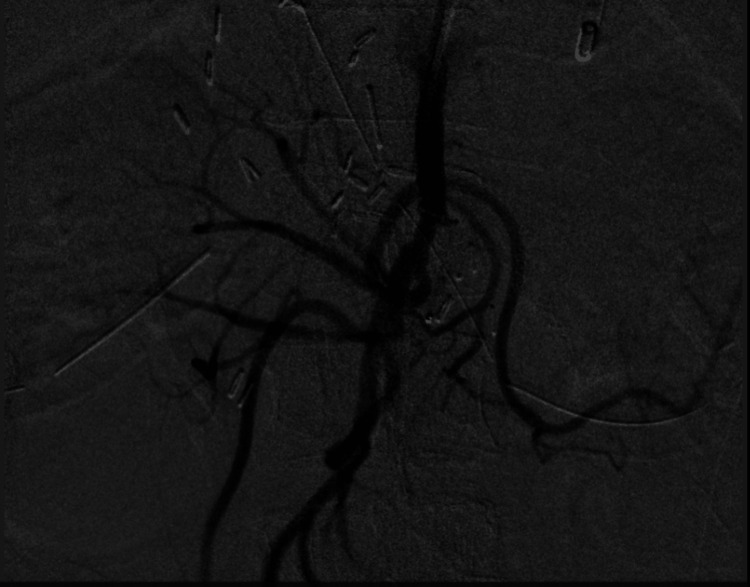
Post-embolization angiogram showing successful occlusion of the bleeding vessel with coils and no further evidence of contrast extravasation.

Post-embolization angiography demonstrated complete occlusion of the pseudoaneurysm with no residual contrast extravasation (Figure 2). Following embolization, the patient continued to receive vasopressor and ventilatory support in the ICU. Despite aggressive transfusion (a total of 4 units of packed red blood cells, 2 units of fresh frozen plasma, and 1 unit of platelets) and fluid resuscitation, the patient’s hemodynamic instability persisted. Repeat CECT 12 hours later showed no active bleeding or new collections. Given his critical condition, ongoing shock, and poor physiological reserve, re-exploratory surgery was deferred after multidisciplinary discussion. The patient unfortunately succumbed within 24 hours of embolization due to massive hemorrhage-related multi-organ failure.

Final histopathology confirmed moderately differentiated periampullary adenocarcinoma (pT2N1M0) with negative resection margins. There was no evidence of pancreaticojejunostomy leak or intra-abdominal sepsis before the bleeding event.

This case illustrates the catastrophic nature of delayed PPH, emphasizing that not all postoperative bleeds labeled as “sentinel” are minor. Prompt imaging, early angiography, and endovascular intervention remain critical to management. However, the prognosis remains guarded when massive hemorrhage and systemic instability coexist, even after technically successful embolization.

## Discussion

PPH is a major surgical complication associated with considerable mortality and morbidity, particularly in its delayed form (DPH). Although uncommon, delayed PPH remains one of the leading causes of late postoperative deaths following pancreatic resections such as pancreaticoduodenectomy (Whipple’s procedure) and distal pancreatectomy. Recent single-center and multi-institutional studies report mortality rates of up to 40-50%, underscoring its clinical gravity [6,7].

In our case, a 52-year-old male (height 172 cm, weight 74 kg, BMI 25.0 kg/m²) developed a single episode of massive delayed hemorrhage on postoperative day 6, which was promptly managed with emergency endovascular embolization. There was no recurrent bleeding episode prior to the patient’s demise. Hence, the earlier mention of “recurrent” or “sequential” interventional procedures has been corrected to reflect the true course, a single angiography and embolization performed immediately after the bleed.

In contemporary practice, contrast-enhanced CT angiography (CECT-A) is recommended as the first-line diagnostic test in cases of sentinel or overt bleeding following pancreatic surgery, as it helps localize the site and etiology of bleeding with high sensitivity [8]. Catheter angiography is then used for definitive diagnosis and therapeutic intervention. In our case, CECT demonstrated a pseudoaneurysm of the GDA stump, confirmed by digital subtraction angiography (DSA), which was successfully treated with coil embolization.

The GDA stump remains the most common bleeding source (approximately 60% of cases), followed by the common hepatic artery, splenic artery, and superior mesenteric artery branches [9]. The etiology in delayed PPH often involves vascular erosion secondary to pancreatic fistula or intra-abdominal infection; however, in this case, there was no biochemical or imaging evidence of pancreaticojejunostomy leak or abscess formation. The drain output, which had previously been serous (80-100 mL/day), became altered and blood-stained on the day of the bleed,indicating secondary hemorrhagic contamination rather than persistent leakage.

Transcatheter arterial embolization (TAE) using coils, particles, or liquid embolics is now widely accepted as the first-line treatment for delayed PPH, achieving technical success rates exceeding 80% in several series [10-12]. However, mortality remains high due to the patient’s unstable physiological status and associated complications rather than failure of the procedure itself. Covered stent (stent-graft) placement may be considered in selected cases involving major vessels, such as the common hepatic artery, particularly when maintaining arterial perfusion is crucial [13,14].

A multidisciplinary hemorrhage team typically includes hepatopancreatobiliary (HPB) surgeons, interventional radiologists, anesthesiologists, and critical care specialists. Such coordinated teams enable rapid decision-making, expedite transfer for imaging or intervention, and optimize patient resuscitation and monitoring. Early activation of this team is now considered a best-practice model in high-volume pancreatic centers [15].

Our case reinforces several key lessons: (1) delayed PPH, although rare, can be catastrophic and may present abruptly without prior warning; (2) prompt CT angiography followed by timely endovascular management remains the cornerstone of diagnosis and treatment; and (3) despite technically successful embolization, patient outcome is highly dependent on pre-existing comorbidities, physiological reserve, and timing of intervention. Early recognition and a coordinated, multidisciplinary approach remain essential to improving survival in this life-threatening condition.

## Conclusions

DPH is a rare but devastating complication, carrying high morbidity and mortality even with modern perioperative care. Our case underscores the critical importance of recognizing sentinel bleeding as an early warning sign, warranting urgent angiographic evaluation and interventional radiology (IR)-guided embolization. IR offers a minimally invasive and effective alternative to high-risk surgical re-exploration, with the dual advantage of diagnosis and therapy. However, outcomes remain poor when complicating factors such as pancreatic leak, infection, or sepsis are present, as these significantly increase the likelihood of rebleeding despite technically successful embolization.

This highlights the necessity of close postoperative surveillance, rapid referral, and a coordinated multidisciplinary approach involving surgeons, interventional radiologists, and intensive care specialists. Looking ahead, standardizing treatment protocols, developing rapid-response hemorrhage teams, and refining endovascular strategies, such as covered stent-graft placement, may further improve outcomes. Vigilance, early recognition, and timely intervention remain the cornerstone of DPH management.
